# Development and Validation of an Interpretable Machine Learning Model for Inpatient Fall Risk Using Electronic Health Record Data

**DOI:** 10.3390/nursrep16080283

**Published:** 2026-08-13

**Authors:** Siti Zubaidah Mordiffi, Xiujuan Guo, Mien Li Goh, Kee Yuan Ngiam, Neng Wei Wong, Jenny Chua, Mohammad Shaheryar Furqan, Han Shi Jocelyn Chew

**Affiliations:** 1 Nursing Department, National University Hospital, National University Health System, Singapore 119074, Singapore; siti_zubaidah@nuhs.edu.sg; 2 Division of Biomedical Informatics, Department of Surgery, Yong Loo Lin School of Medicine, National University of Singapore, Singapore 119228, Singapore; guo_xiujuan@nuhs.edu.sg (X.G.); m.shaheryar.furqan@nhghealth.com.sg (M.S.F.); 3 Department of Surgery, National University Hospital, National University Health System, Singapore 119074, Singapore; surnky@nus.edu.sg (K.Y.N.); wong_neng_wei@singhealth.com.sg (N.W.W.); 4 Division of Oncology Nursing, National University Cancer Institute Singapore, National University Health System, Singapore 119074, Singapore; 5 Alice Lee Centre for Nursing Studies, Yong Loo Lin School of Medicine, National University of Singapore, Singapore 117599, Singapore; jocelyn.chew.hs@nus.edu.sg

**Keywords:** artificial intelligence, falls, adverse events, machine learning, decision tree, support vector machine, fall risk prediction

## Abstract

**Background**: Falls are the most common hospital-acquired adverse event, leading to extended hospitalization, loss of independence, disability, and premature death. Routine fall risk assessments are time-consuming, even with limited factors. An AI-derived fall prediction model can provide more comprehensive and comparably accurate risk predictions quickly and as often as needed. **Objective**: To develop and validate a fall prediction model for fall risk in adult inpatients. **Methods**: Patient records from 2016 were extracted from the adult inpatient database, including information from the Electronic Inpatient Medication Records, SAP, and Hospital Incident Reporting System. The sample consisted of 1506 cases (1:5 faller to non-faller). The fall prediction model was trained using the following four variables: demographics, diagnosis, medications, and surgery. Data sources included the hospital’s data repository, integrating admission/discharge, pharmacy, laboratory, and incident reports. **Results**: The support vector machine model performed best among all tested models, achieving an AUC of 0.803, recall of 0.816, and precision of 0.440. In the validation cohort (978 patients: 163 fallers and 815 non-fallers), the fall prediction model demonstrated moderate-to-good discrimination (AUC 0.79), with accuracy of 0.67, sensitivity of 0.46, and specificity of 0.86. Compared with the nursing four-item fall risk assessment, which showed lower discrimination (AUC 0.65, accuracy 0.65, sensitivity 0.58, specificity 0.72), the fall prediction model had better specificity and overall discrimination, though the nursing tool was more sensitive in identifying fallers. **Conclusions**: The fall prediction model using demographics, diagnoses, medication, and surgery data predicts falls risk effectively. It enables timely, accurate risk assessments and supports preventive interventions, saving nurses’ time for direct patient care.

## 1. Introduction

### 1.1. Fall Issues

Falls are the second leading cause of accidental death among adults over 60, contributing to prolonged hospitalization, loss of independence, disability, and increased healthcare costs [1]. Despite the routine use of manual fall risk assessment tools, falls during hospitalization remain a persistent challenge, leading to poor patient outcomes, legal implications, and workflow disruptions [2,3].

### 1.2. Fall Prevention—Assessment Tool

Globally, nurses typically conduct regular fall risk assessments using tools such as the Hendrich High-Risk Fall Model [4] and the Morse Fall Scale [5]. While these manual assessments are standard practice, they are time-consuming, labor-intensive, and rely on fixed criteria that may not capture the full complexity of individual patients’ risk profiles [6]. At our hospital, nurses use a four-item assessment tool—evaluating confusion, dizziness, altered elimination, and difficulty with mobility—to screen for fall risk [7]. Assessments are performed upon admission, daily, and whenever a patient’s condition changes, adding to the already high workload of nursing staff. Furthermore, although these four risk factors are common in the local population, they may not adequately represent the diverse risk profiles of all inpatients, limiting the tool’s generalizability.

### 1.3. Proposed Strategy—AI-Enabled Fall Prevention Assessment

To ensure patient safety and enhance the quality and efficiency of care, we propose the development and validation of an artificial intelligence (AI)-enabled fall prediction model. This model is designed to assist nurses in predicting patients’ fall risk in a timely manner using updated, real-time clinical data. The long-term goal is for the fall prediction model to be fully integrated with the hospital’s electronic medical record (EMR) system (EPIC), operating independently as part of routine clinical workflow.

Recent advances in AI and the integration of electronic health records (EHRs) have enabled the development of predictive models that can rapidly and accurately assess fall risk. Studies demonstrate that AI-assisted tools can outperform traditional assessments, improve patient outcomes, and reduce costs [8,9,10,11,12]. However, most existing models have limited generalizability and are not fully integrated into routine clinical workflows.

Several studies have examined the development of AI-assisted fall prediction models specifically for inpatient acute care settings (Table 1) [13,14,15,16,17]. A meta-analysis confirmed that AI-powered EHR systems can accurately predict inpatient fall risk, suggesting that practical hospital applications may be forthcoming [18]. In addition, various AI models have been developed for targeted patient populations. For instance, one research team created a system for patients with hypertension and later refined their algorithm for ophthalmology patients by incorporating recent deterioration in visual acuity as a predictive factor [19,20]. In a separate study, XGBoost—a machine learning method—was applied to large-scale electronic health records to estimate fall risk in older adults [21]. These developments highlight the expanding application of AI to enhance fall risk prediction across diverse clinical settings.

This study addresses these gaps by developing and validating an AI-enabled fall prediction model designed to operate in real time within the hospital’s electronic medical record system. By leveraging comprehensive, up-to-date clinical data, the model aims to provide timely and individualized risk assessments for adult inpatients.

### 1.4. AIM

This study aims to develop and validate a fall prediction model for fall risk in adult inpatients.

## 2. Methods

### 2.1. Study Design and Setting

This was a retrospective observational study of electronic medical records to derive a predictive model to predict fall risk. It was conducted in a 1200-bed academic tertiary hospital.

### 2.2. Ethical Considerations

The study was approved by the National Healthcare Group Domain Specific Review Board (DSRB) (reference 2019/01040). Approval to access and extract de-identified data was obtained from the National University Hospital (NUH) Medical Affairs Department through the Academic Information Office.

### 2.3. Source of Data

Our dataset was retrieved from the 2016 inpatient database of the Singapore National University Hospital (NUH) (referred to as a “DataMart”), which includes information from the Electronic Inpatient Medication Records (eIMRs), SAP (Systems Applications and Products) and the Hospital Incident Reporting System (HIRS).

The eIMR includes data on the medications prescribed for each patient. SAP contains data on each patient’s demographic profile, admission and discharge records, diagnosis, surgery, and procedures. HIRS includes data on fall incidence, admission time, fall time, and patient discharge time. Based on the HIRS database, there were an estimated 251 cases of falls in adults (≥20 years old) in 2016. Data extraction of the non-fall instances was based on unique cases instead of unique patients to prevent missing out on multiple falls by the same person. To build a dataset that includes fall and non-fall cases in a 1:5 ratio, we extracted 251 cases of falls from 2016 and, using a random permutation technique, extracted another 1255 records from 101,410 cases of non-fall to form a matched control group for the fall cases (Table 2). A ratio of 1:5 was chosen to take into account the disproportionately higher numbers of non-fallers compared to fallers.

Following approval, fall-related data were extracted by a designated third party and stored in an encrypted file on a corporate-issued computer, accessible only via the institution’s secure intranet using authorized login credentials. Subsequently, SAP-related data were retrieved by a separate third party from the Discovery AI platform. All data were de-identified prior to analysis to ensure patient confidentiality and data security throughout the study.

**Figure 1:**
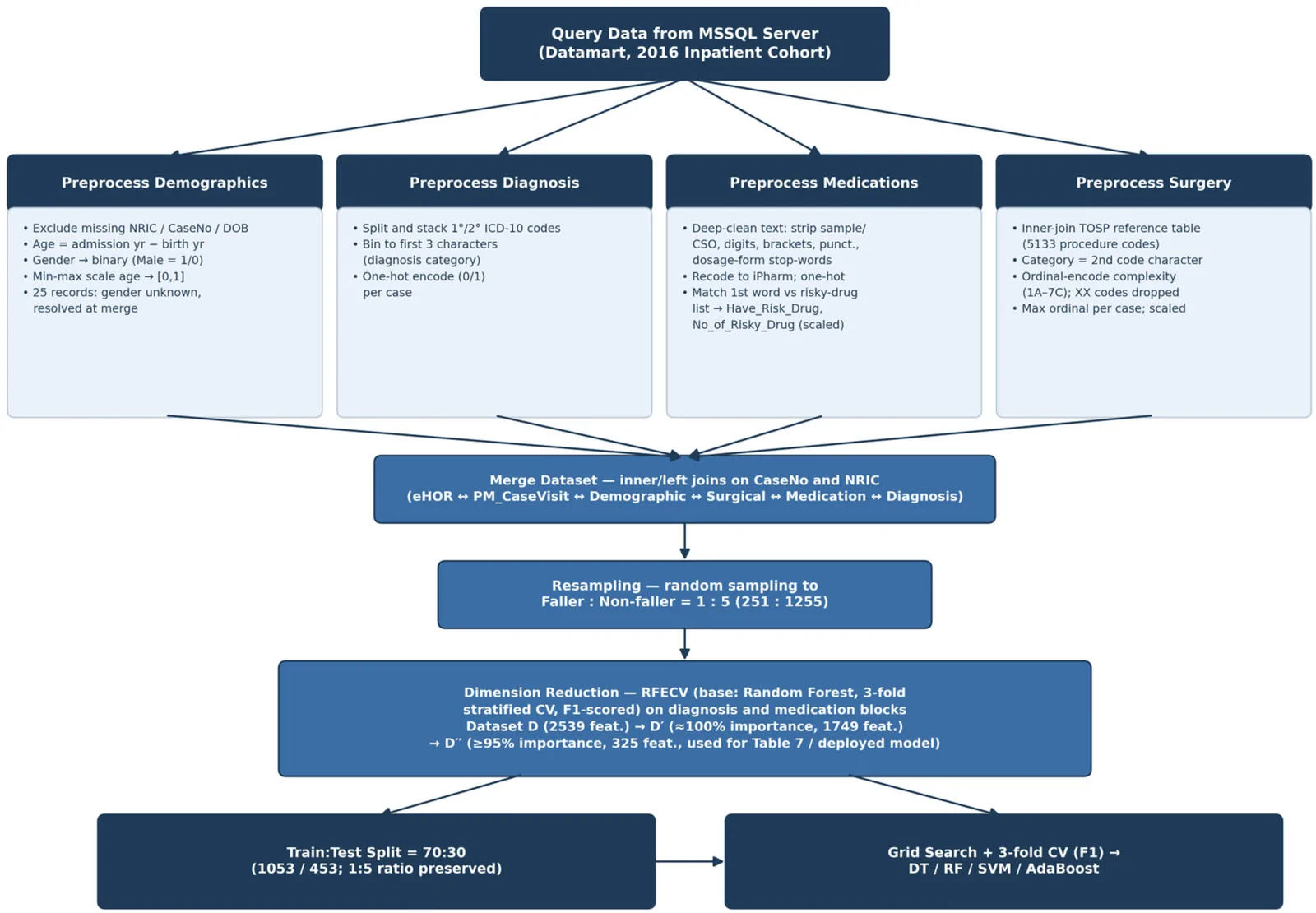
Overview of machine learning model development. Notes: MSSQL = Microsoft structured query language server; HIRS/eHOR = Hospital Incident Reporting System; TOSP = Table of Surgical Procedure; RFECV = Recursive Feature Elimination with Cross-Validation.

An overview of the development of the machine learning model is shown in Figure 1 below.

### 2.4. Participants

Participants for model development were adult inpatients aged 20 years or older, admitted to the study hospital during the calendar year 2016. Participants for temporal validation were adult inpatients aged 20 years or older admitted between June 2022 and June 2023. No other exclusion criteria were applied beyond the removal of records with missing NRIC, case number, or date of birth, described under Missing Data below.

### 2.5. Outcome

**Figure 2:**
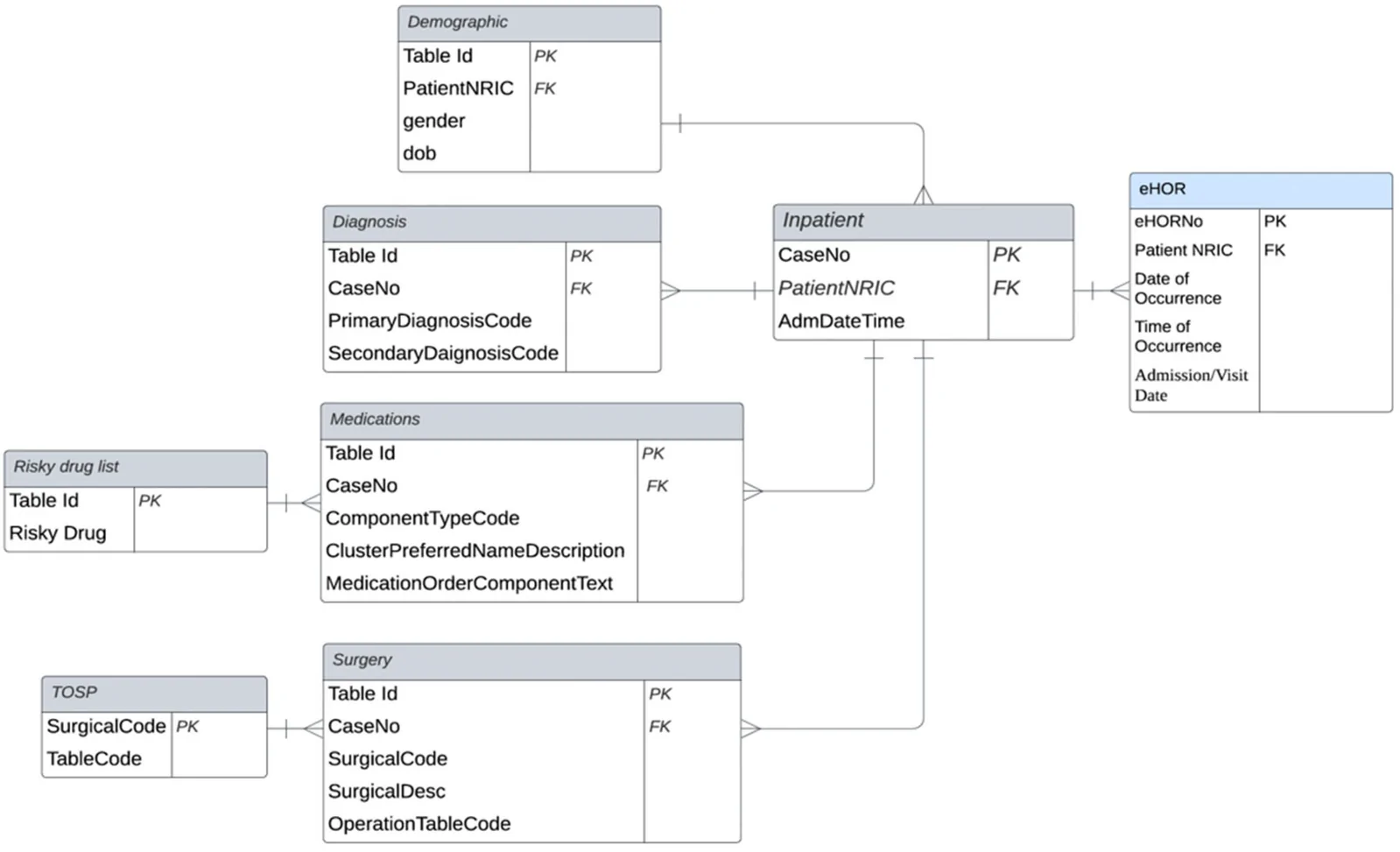
Entity relationship diagram (ERD) of the final merged dataset. Note: PM—patient management; No—number; NRIC—National Registration Identity Card; HIRS—Hospital Incident Reporting System (HIRS); TOSP—Table of Surgical Procedure; PK—primary key; FK—foreign key.

The HIRS was inner-joined with patient management case visits based on National Registration Identity Card (NRIC) and inpatient fall event (labeled as 1 = high fall risk) to identify fallers. For fallers with multiple fall accidents in one patient stay, namely in the case number, only the earliest fall incident was kept in the faller dataset. Non-faller status was determined at the patient level. Once a patient’s National Registration Identity Card was associated with any fall incident recorded in the HIRS, all of that patient’s other admissions were excluded from the dataset entirely, regardless of whether those admissions occurred before or after the recorded fall, so that no patient contributed more than one case and no patient contributed records to both the faller and non-faller groups. Only cases belonging to patients with no fall incident recorded in the HIRS were retained as non-faller, labeled as 0, low risk of fall. This main table was inner-joined with demographic data to merge different datasets into one final dataset. Diagnosis, medications, and procedure data were left joined with the main table based on the case number and NRIC. Patients with incomplete medication and procedure information had those values replaced by 0. Figure 2 illustrates the entity relationship diagram (ERD).

Blinding of outcome assessment was not applicable, as this was a retrospective study using routinely collected electronic health record and incident report data rather than a prospective assessment.

### 2.6. Predictors

#### Data Cleaning, Preprocessing, Feature Engineering, and Exploration

To identify the relevant predictors of falls, we first extracted all the information on the patient’s demographic profile (i.e., age and sex), diagnoses, fall incidence reports (i.e., occurrence time, patients’ identification number), medications (i.e., list of medicines that may lead to fall), and surgical procedures (i.e., surgical procedure category and procedure scale) received. All the medical data were preprocessed to generate consistent item names. For example, all medication data were recoded using the iPharm database convention; diagnoses were recoded using the International Classification of Diseases (ICD10) codes, surgical procedures were recoded using the Table of Surgical Procedure (TOSP) codes. Missing values and outliers were removed for feature engineering. The final dataset contained 1506 records. Categorical data such as diagnoses, surgery, and medications were recoded into binary variables. For diagnoses, we binned the diagnosis by extracting the first 3 letters and regarded them as diagnosis categories—consistent with the hierarchical structure of the International Classification of Diseases, 10th Revision, in which the first three characters already correspond to a specific, clinically meaningful diagnostic block. For example, the block C30 to C39 corresponds specifically to malignant neoplasms of the respiratory and intrathoracic organs, and A00 to A09 corresponds specifically to intestinal infectious diseases. This level of the classification therefore provides sufficient diagnostic granularity for feature construction without extending to the full alphanumeric code, which would have produced a very large number of sparsely populated categories given the sample size available. Scale data such as age and ranking of surgical complexity (1A to 7C) were converted into numeric data and then rescaled to a range of 0 to 1. This information was then transformed into a sparse matrix for machine learning modeling using different model techniques and model hyperparameters’ fine-tuning.

Medication names were deep-cleaned prior to iPharm recoding as follows: raw order text was case-folded, leading “sample”/“CSO” tokens and leading digits were removed, bracketed/parenthetical content and punctuation were stripped, and a fixed stop-word list of dosage-form and formulation terms (e.g., tablet, capsule, infusion, gel, cream, injection, solution, and syrup) was removed before one-hot encoding. Each cleaned medication name was additionally matched, on its first word, against a curated high-risk (“risky”) drug reference list, generating the following two engineered features: Have_Risk_Drug (binary) and No_of_Risky_Drug (count, subsequently min–max scaled).

Surgical procedure codes were inner-joined against the Table of Surgical Procedure (TOSP) reference table (5133 procedure codes) on an exact code match; the surgical category was extracted as the second character of the procedure code, and the corresponding TOSP complexity ranking (1A to 7C) was ordinal-encoded and min–max-scaled alongside age. For cases with more than one surgical procedure, the maximum (most severe) ordinal value across that case’s procedures was retained as the single surgical-severity feature for that case. Surgical records carrying a placeholder “XX” Operation Table Code (i.e., an invalid or non-substantive entry) were dropped prior to TOSP merging.

### 2.7. Missing Data

Case-level attrition in the faller-identification pathway can be reported explicitly as follows: of 457 fall incidents recorded in the Hospital Incident Reporting System (eHOR) for 2016 (424 unique patients by NRIC), 287 were matched to an inpatient admission where the fall date/time fell within the admission–discharge window and were retained as confirmed inpatient fallers; 939 records had a fall time outside the matched admission’s window and were excluded. Of further 89 faller patients who could not be matched to any inpatient record, 26 were identified in outpatient records and 63 could not be located in either inpatient or outpatient records and were excluded entirely. Subsequent restriction to age ≥ 20 years and retention of only the earliest fall event per multi-fall patient yields the 251 faller cases reported in Table 2. Gender was recorded as unknown for 25 patients, resolved during the demographic merge step.

### 2.8. Sample Size

Non-faller controls were selected by simple random permutation sampling (i.e., without explicit matching on demographic confounders such as age or sex); age and sex were instead retained as explicit predictor variables so the classifier could adjust for these factors directly, consistent with the principle that matching is not required and can itself introduce selection bias, when confounders are entered as covariates in the analytic model [22]. The 1:5 faller-to-non-faller sampling ratio reflects the established finding that the statistical efficiency gained from adding further controls per case diminishes rapidly beyond a ratio of approximately 4:1–5:1 [23].

### 2.9. Statistical Analysis Methods

Full detail on the statistical analysis methods used to build and select the fall prediction model, including predictor handling, model building procedures, predictor selection through recursive feature elimination, hyperparameter tuning, and internal validation, is presented in Section 3.4 and Section 3.5, alongside the reporting of model performance.

## 3. Results

This study involved 1506 patients, including 251 fallers and 1255 non-fallers. After merging tables, the final dataset D (Table 3) had 2539 variables, including 1202 medications, 1320 diagnoses, 15 surgery procedures, and two demographics variables. Data exploration was performed on demographics, including age and gender, and surgeries for pattern mining. In addition, a recursive feature elimination technique was performed on diagnoses and medications.

### 3.1. Demographics—Age and Gender

Table 3 shows an overview of the demographic distributions of all inpatients, fallers, and non-fallers in 2016.

The mean age of all patients was 54.86 years (SD = 18.61). Fallers (M = 61.67, SD = 16.27) were generally older than non-fallers (M = 54.84, SD = 18.61). The proportions of males and females among non-fallers were 51% and 49%, respectively. More male patients had fall incidences (62% males vs. 38% females).

### 3.2. Surgery

**Figure 3:**
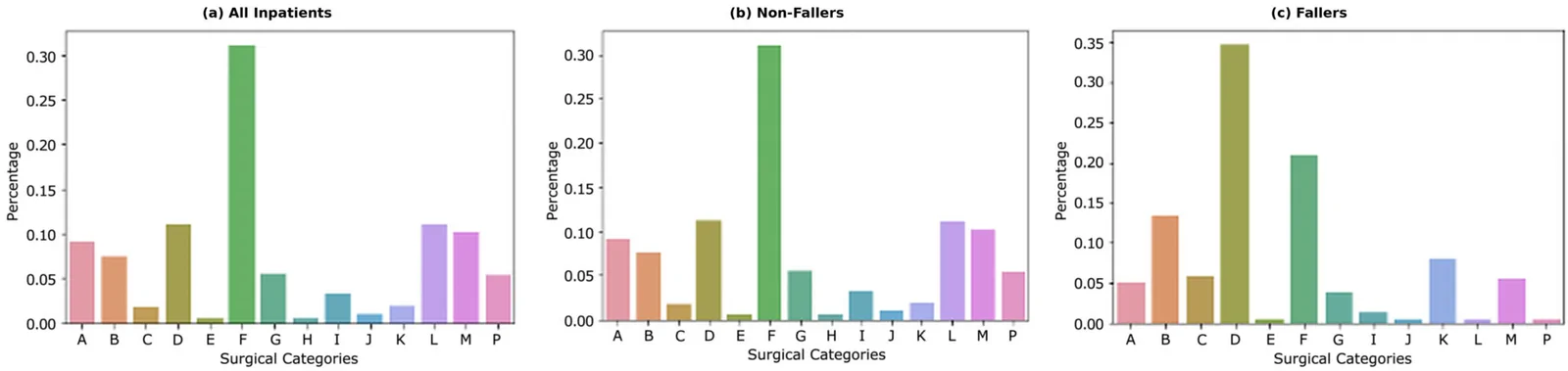
Surgery (surgical categories) distributions for all inpatient patients, non-fallers, and fallers in the year 2016. Notes: Surgical categories are defined according to the Table of Surgical Procedures (TOSP): A = Integumentary (Skin and Breast); B = Musculoskeletal; C = Respiratory; D = Cardiovascular; E = Hemic and Lymphatic; F = Digestive; G = Urinary; H = Male Genital; I = Female Genital; J = Endocrine; K = Nervous; L = Eye; M = Ear, Nose and Throat; P = Obstetric.

Figure 3 shows the surgical category distributions among all inpatients, non-fallers, and fallers. In all inpatients, surgical category F (Digestive) has the highest percentage among 14 categories, followed by categories D (Cardiovascular) and L (Eye). In non-fallers who had undergone surgeries, the highest proportion was under category F, followed by categories L and D. However, none of the fallers underwent surgeries belonging to category H (Male Genital). Instead, fallers had the highest proportion of surgeries under category D, followed by surgeries under category F and then category K (Nervous).

### 3.3. Medications and Diagnoses

As the number of variables, which included 1320 diagnoses and 1204 medications, was twice as many as the data points (*n* = 1506), we faced the risk of a phenomenon known as the “curse of dimension” where the number of observation configurations decreased as the dimension of features increased. Therefore, recursive feature elimination (RFE) was applied separately to the following two largest features: medication and diagnosis.

### 3.4. Recursive Feature Elimination (RFE)

RFE technique was applied to variables in the aspect of diagnoses and medications, the total number of which was 1320 and 1204, respectively. RFE was implemented as scikit-learn’s cross-validated RFECV, using random forest as the base estimator with its internal impurity-based feature importance ranking, 3-fold stratified cross-validation, and the F1 score as the ranking metric. Random forest was selected because regression-family approaches (multiple linear regression, logistic regression, and linear discriminant analysis) cannot be used for RFE when candidate predictors exceed the number of samples, as here; random forest instead supports post hoc pruning and provides a usable importance ranking, though its scores can be diluted by multicollinearity among correlated features. The random forest base estimator was itself grid-search-tuned separately for each feature domain (3-fold stratified CV, F1-scored) as follows: for diagnoses, the selected configuration was n_estimators = 20, criterion = entropy, max_depth = 5, max_features = log2, class_weight = {0:1,1:5} (CV F1 = 0.510); for medications, n_estimators = 60, criterion = gini, max_depth = 8, max_features = sqrt, class_weight = {0:1,1:5} (CV F1 = 0.608). This technique selected 707 diagnoses and 1025 medications (Table 4). The sum of feature importance was close to 1 (99.9%). The 707 diagnoses and 1025 medications were merged with 15 surgical procedures and two demographic variables to form a dimension-reduced dataset of 1749 features (dataset D′).

To further reduce the dimensions, we selected the most important variables (score of ≥95% of the overall feature importance). After removing the least important variables, 92 diagnosis variables and 216 medication variables remained (Table 4a), with a sum of remaining feature importance >95% (dataset D′). These remaining variables were then merged with 15 surgical procedures and two demographic variables, adding up to a total of 325 features.

Our final dataset had 1506 records, including 251 faller records and 1255 non-faller records. We then split the data into a training and a testing dataset in a 7:3 ratio. In the training and testing datasets, the fallers and non-fallers remained in a ratio of 1:5. In the training dataset, there were 175 fallers and 878 non-fallers and 1053 data points (records). In contrast, in the testing dataset, there were only 76 fallers, 377 non-fallers, and 453 data points (Table 4b), with each data point having 2539 predictor variables and one target variable.

To select the best algorithm for this dataset (dataset D), we adopted six modeling techniques, namely logistic regression (LR), decision tree classifier (DT), random forest decision tree classifier (RFDT), support vector machine classifier (SVM), gradient boost decision tree classifier (GBDT), and AdaBoost decision tree classifier (AdaBoost). Three (DT, RFDT, and SVM) out of these six models were selected as our baseline models based on the evaluations of the Confusion Matrix, F1 score, precision, recall, and area under the receiver operating characteristic curve (AUC) score. Decision tree is a supervised learning method used mainly for classification problems, where each internal node represents a feature, each branch represents a decision rule, and each leaf node represents an outcome [24]. Random forest (RF) is an ensemble learning method that uses multiple independent decision trees to classify samples into various categories [25], from which it generates a final aggregate score of the category with the highest vote. SVM is a strategy to classify data by drawing and deciding on the most suitable hyperplane to separate the data [26]. These three baseline models were tested on our dimensionally reduced dataset (datasets D′ and D″). The grid search method was performed to identify the best hyperparameters of all three models. We tested different sets of hyperparameters for each model on the training dataset (1053 data points) within grid search methods, in conjunction with the F1 score and 3-fold cross-validation, to identify each model’s best set of hyperparameters. Models with the best set of hyperparameters were then evaluated in terms of F1 score, precision, recall and specificity values on our training dataset (453 data points). In addition, AUC was also used as one of the indicators to evaluate the model overall performance.

### 3.5. Machine Learning Model Performance

Our goal in rating the best model was to pursue the maximum recall (sensitivity) with acceptable specificity. Therefore, the SVM classifier, DT classifier, and RFDT classifier were selected as our baseline models for further experimentation. On the full feature set (Table 5), RFDT in fact achieved a marginally higher AUC (0.807 vs. 0.803) and specificity (0.838 vs. 0.790) than SVM, while SVM achieved the higher recall (0.816 vs. 0.776); consistent with the recall-first selection goal stated above, SVM was retained despite not leading on every metric.

All three models’ performances were similar in dataset D (Table 5), D′ (Table 6), and D″ (Table 7). The recall values were between 0.7 and 0.8, precision values were between 0.4 and 0.5, and AUC_ROC (under the curve receiver operating characteristics) values were between 0.7 and 0.8. This gave insights that the 325 variables in dataset D″ could represent both dataset D and dataset D′ well. Thus, the model results based on dataset D″ were then compared with the research results of two manual assessment tools (Table 7).

With the best parameter combination for each model using the same features, the best-performing model in predicting falls was SVM (AUC = 0.800; recall = 0.803; specificity = 0.798; and precision = 0.445) (Table 7).

### 3.6. Validation Results

We validated our fall prediction model using data from adult inpatients aged 20 and above who were admitted between June 2022 and June 2023. We focused on the following two groups: patients who experienced a fall and all patients admitted during this period, using a faller to non-faller ratio of 1:5. Among the 163 real fall cases, the model correctly identified 55 patients as high risk, while nurses also assessed them as high risk. It also predicted 21 patients as high risk when nurses did not and missed 40 cases that nurses did identify as high risk. In 47 cases, both the model and nurses did not assess the patient as high risk. Looking at all 59,963 admissions, the model and nursing assessments agreed on high risk for 4905 patients (including 55 fallers), while the model alone flagged 2878 patients (21 fallers), and nurses alone flagged 11,490 patients (40 fallers). Both agreed on low risk for 40,690 patients (47 fallers).

**Figure 4:**
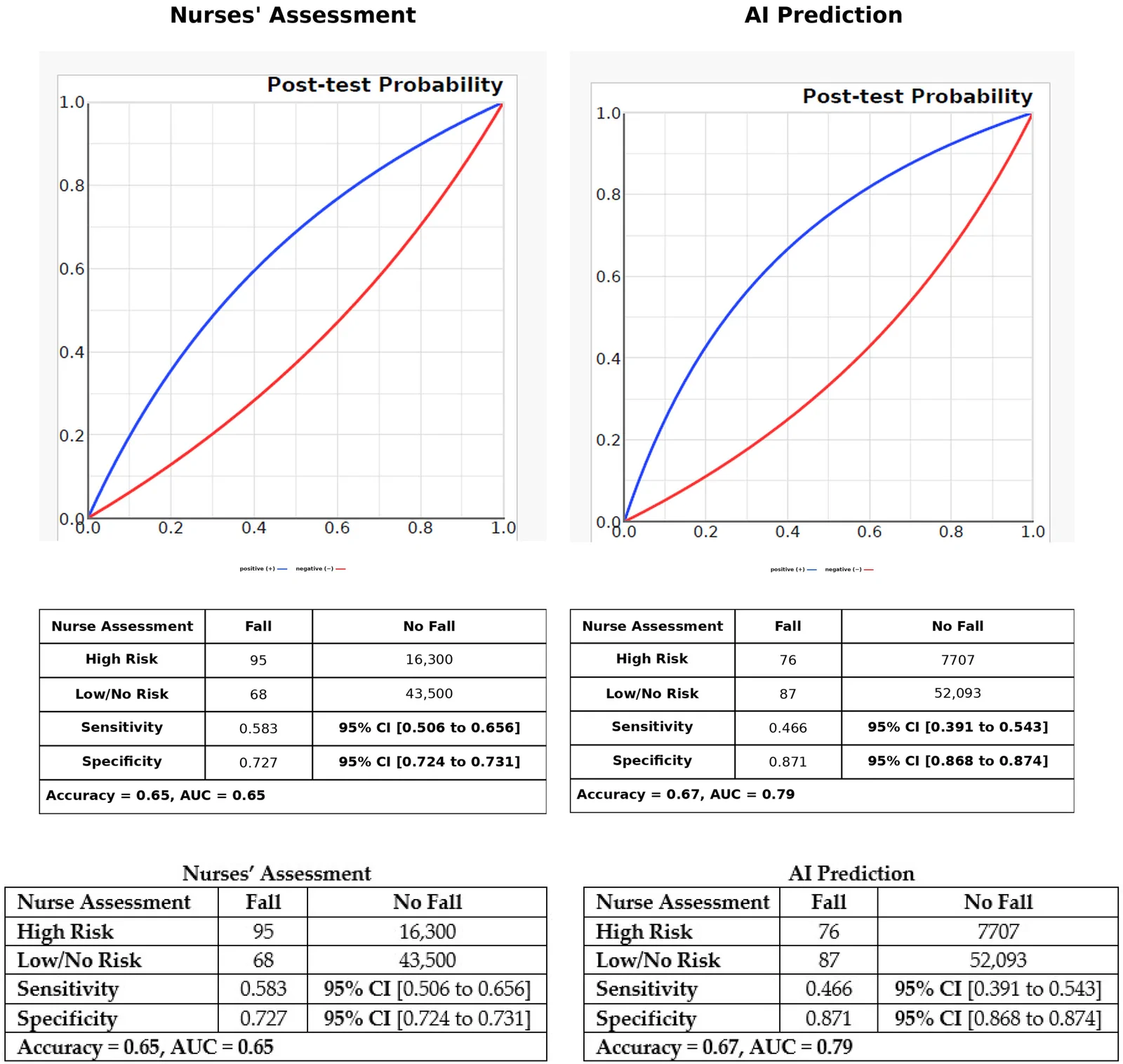
Accuracy of nurses’ assessment and AI prediction. N = 59,963 (adult inpatient June 2022–June 2023).

When the validation set with a faller to non-faller ratio of 1:5 (163 fallers, 815 non-fallers, total 978 cases), the AI fall model achieved a sensitivity of 0.46 and a specificity of 0.86, with 76 true positives, 87 false negatives, 111 false positives, and 704 true negatives. The area under the receiver operating characteristic curve (AUC) for the fall prediction model was 0.79 and accuracy of 0.67. In comparison, the nursing four-item fall risk assessment achieved a sensitivity of 0.58 and a specificity of 0.72, with 95 true positives, 68 false negatives, 224 false positives, and 591 true negatives, yielding an AUC of 0.65 and accuracy of 0.65. These results indicate that while the nursing assessment showed higher sensitivity, the fall prediction model provided higher specificity and overall discrimination as reflected by a superior AUC. Some challenges in validation included the time gap between the training data (2016) and the validation data (2022), differences in data features like medication names, and the need to adjust model thresholds for the new data (Figure 4).

### 3.7. Model Interpretability

The linear kernel of the support vector machine produces a directly interpretable weight vector, in which the sign and magnitude of each coefficient indicate whether a feature is associated with a higher or lower predicted fall risk. The complete set of 325 coefficients, ranked by magnitude, is provided in Appendix A, Table A1, with the coefficients for the surgical category and demographic feature blocks in Table A2, and the corresponding random forest feature importance rankings for diagnosis and medication predictors in Table A3 and Table A4.

The two largest coefficients in the model correspond to the diagnoses R55, syncope and collapse, and S09, other or unspecified injuries of the head. Syncope is a well-recognized direct precipitant of falls, and its prominence in the model is consistent with established clinical understanding of fall mechanisms. S09 may reflect a related but distinct pathway as follows: a recorded head injury can represent either a consequence of a prior fall or an independent marker of frailty and susceptibility to trauma, and this diagnosis should therefore be interpreted as a signal of accumulated risk rather than as a single causal factor.

Several of the next largest coefficients correspond to medications with recognized links to fall risk. Sedating and psychoactive agents, including quetiapine, haloperidol, alprazolam, and hydroxyzine, are established fall-risk-increasing drugs that act through sedation, psychomotor slowing, and orthostatic hypotension. Laxative and bowel-care agents, including lactulose, bisacodyl, and sennosides, are also prominent among the top coefficients; these may act as a proxy for altered elimination status, one of the four items in the existing manual nursing assessment tool, and for the associated increase in nocturnal toileting and unassisted mobility that this entails. Together, these findings indicate that the model’s most influential predictors have biologically and clinically plausible relationships to fall risk, supporting its interpretability alongside its predictive performance.

Model coefficients for the linear kernel SVM, together with random forest feature importance rankings, are reported in Section 3.7, with the full tables provided in Appendix A, Table A1, Table A2, Table A3 and Table A4. The linear SVM produces a directly interpretable weight vector, in which the sign and magnitude of each coefficient indicate whether a feature is associated with higher or lower predicted fall risk. Clinicians should interpret these weights as indicators of the relative contribution of each variable within the model rather than as independent, causal, or diagnostic thresholds for individual patients. We further acknowledge an important limitation as follows: several clinical variables in this dataset, such as related medications or co-occurring diagnoses, are likely to be correlated with one another. Coefficients from a linear model estimated on correlated predictors can be unstable and may not isolate the independent effect of each variable, so individual coefficient values should be interpreted with caution rather than as precise, standalone estimates of risk contribution.

## 4. Discussion

### 4.1. Principal Results

Our results showed that our fall prediction model using SVM built upon age, gender, diagnoses, fall incidence reports, medications, and surgical procedures outperformed the manual fall risk assessment tool currently used by nurses in NUH (AUC = 0.657; sensitivity = 0.81, and specificity = 0.44) [7] and the Hendrich Fall Risk Model (AUC = 0.667, sensitivity = 0.75 and specificity = 0.51). These results suggest that the fall prediction model may have the potential to complement the current manual fall risk assessment. However, this study evaluated predictive performance only. It did not assess clinical implementation, workflow integration, patient outcomes, or nursing workload, and any conclusions regarding these aspects would require prospective implementation studies [16]. A similar study approach using machine learning methods to determine hospitalized patient at risk to fall included 38 predictor variables comprising patient characteristics, admission information, assessment information, clinical data, and organizational characteristics, used classification tree, bagging, random forest, and adaptive boosting methods compared with the nurses’ fall risk assessment using the Morse Fall Scale. The machine learning models outperformed the scale, supporting and enhancing nurses’ ability to predict and care for patients at risk of falls [15]. However, comparisons across studies were challenging due to differences in populations, features, and machine learning models; still, gender, diagnoses, and medications were commonly included features [16,27].

A plausible mechanism for this potential benefit can be described. The fall prediction model draws on a broader range of routinely recorded clinical data, including diagnoses, medications, and surgical history, than the four-item nursing assessment, which depends on the nurse directly observing and interacting with the patient. When a nurse is caring for a patient for the first time, it can be difficult to judge, for example, whether that patient has altered or abnormal elimination status without an extended period of observation. However, the presence of continence products or diaper orders already recorded in the electronic health record may indicate altered elimination status or a need for assisted activities of daily living. By surfacing contributing factors that are already present in the electronic record, the model may assist nurses in reaching an informed assessment more quickly, particularly for patients who are not yet well known to them, supporting rather than replacing clinical judgment. This describes a plausible mechanism of benefit rather than a demonstrated outcome, since this study did not directly measure decision-making speed, nursing workload, or patient outcomes.

Although the simplified four-item fall risk assessment tool was derived from a multitude of global research over the years, and it indeed provides a simple application, it requires nurses to perform it on every patient daily, which is time-consuming. In addition, this simplified assessment tool may not be able to cover complex contributing factors to predict the risk of falls accurately. Thus, we developed a fall prediction model that takes in multiple significant factors and adjusts them to Singapore’s local context to generate accurate results to support clinical decisions in a timely manner. Additionally, if validated through prospective implementation research, an automated fall risk assessment integrated into the electronic medical record system may support nurses during the fall assessment process. This reflects Singapore’s commitment to transforming healthcare through a data-driven approach, supported by a robust artificial intelligence and digital ecosystem to achieve excellence in healthcare [28].

Our validation results are consistent with the recent literature demonstrating the potential of AI-based models to enhance fall risk prediction in hospital settings [29,30]. A 2025 multicenter study across two German hospitals (*n* = 931,726 and *n* = 12,773) found that fall prediction models consistently outperformed rule-based clinical fall risk systems, with SHAP-based explainability analysis identifying key predictors that aligned with established clinical knowledge [29]. Similarly, a 2025 multicenter retrospective study involving five Japanese hospitals reported that gradient boosting models achieved clinically valuable prediction performance, with SHAP analysis consistently identifying low albumin, impaired transfer ability and sedative-hypnotic use as major contributors to fall risk [30]. These results reinforce the potential of our SVM model’s predictor variables, particularly medication categories and diagnoses (including past medical diagnosis), as clinically meaningful risk factors.

The sensitivity–specificity tradeoff observed in our validation findings is consistent with patterns reported in the literature. While our fall prediction model showed superior AUC (0.79) and specificity (0.86) over the nursing four-item assessment tool (AUC 0.65, specificity 0.72), the nursing tool retained higher sensitivity (0.58 vs. 0.46). A 2025 study using machine learning with real-time location system data and electronic medical records achieved a combined AUROC of 0.847, underscoring that integrating dynamic physical activity data alongside clinical variables further improves discrimination [31]. This highlights an important direction for the future development of our model as follows: incorporating mobility- or activity-related features may address the current sensitivity gap. A large retrospective study of 46,695 patients across 17 U.S. hospitals demonstrated that in dynamic models updated at 8 and 24 h intervals, incorporating time-series physio-logical data, the model achieved AUCs as high as 0.87, substantially surpassing static nursing assessment tools [32]. These findings suggest that real-time or regularly updated AI risk scores embedded into electronic nursing workflows could significantly enhance both sensitivity and clinical utility.

Healthcare professionals have been shown to prefer AI tools that provide clear explanations of predictions rather than solely risk scores, as this explanation facilitates trust and supports clinical decision-making [33]. Reporting and communicating these linear weights, alongside emerging explainability methods such as SHAP (SHapley Additive exPlanations) that have been adopted in recent fall prediction models [29,30,32], represents a priority for future iterations of this work to improve nursing acceptance and enhance meaningful clinical use. This is particularly relevant in the Singapore healthcare context, where digital health transformation is a strategic national priority.

The temporal gap between our training data (2016) and validation cohort (2022–2023) represents a real-world challenge that is well-documented in the literature. Changes in patient populations, best practices of treatments, medication formularies, and clinical coding conventions over time can erode model performance, a phenomenon known as dataset shift [34]. Our validation results (AUC 0.79 vs. training AUC 0.80) suggest relative temporal stability of the model, but the observed decline in sensitivity warrants careful attention. A specific, confirmed contributor can now be reported as follows: the 2022–2023 validation cohort was extracted from the newer EPIC electronic medical record (via the EndeavourAI platform), which captures diagnoses in SNOMED CT rather than the ICD-10 convention used for the 2016 training data; because the model’s diagnosis features are ICD-10-binned, a SNOMED–ICD-10 crosswalk step was required to prepare the validation extract. As SNOMED CT’s more granular concept hierarchy does not map cleanly one-to-one onto ICD-10 for every code, incomplete or many-to-one crosswalk coverage is a plausible contributor to reduced information content in the diagnosis-based predictors at validation time. Separately, medications in the 2022–2023 extract were processed using the same cleaning and high-risk-drug matching logic as the 2016 training data, but the underlying medication name text itself differs because it originates from a new source system, which can still reduce feature-matching rates even without any change to the preprocessing code itself. The need for regular recalibration and external validation has been highlighted across multiple AI-based fall prediction studies and should be incorporated into the deployment roadmap for our model within the EPIC electronic medical record system [30,34]. Furthermore, combining AI predictions with clinical expertise has been shown to improve overall prediction accuracy and clinical usefulness, and the complementarity between our fall prediction model and nursing assessment are capturing different signals, suggesting that a hybrid decision support approach may be the optimal path forward [35].

Nurses’ involvement in artificial intelligence research

The significance of this nurse-led study lies in the consistent provision of direct bedside care by nurses. Nurses routinely conduct multiple assessments, with fall assessment among the most critical and prioritized tasks during each shift. Consequently, artificial intelligence (AI) technologies play a pivotal role in managing nurses’ daily workload and supporting clinical decision-making. It is well-established that AI technologies enhance resource management, improve risk assessment, and facilitate clinical decision-making, thereby promoting patient safety [36]. The use of AI tools has been shown to improve discharge support systems, reduce 30-day readmissions, enable deterioration algorithms that shorten the time to contact senior staff and order tests, and predict potential skin injuries. AI predictive algorithms can enhance nursing care performance and expand knowledge to better manage patient conditions. These predictions are often real-time and practical, allowing seamless integration into clinical environments with high demand with healthcare needs [36].

Therefore, nurses should receive training and education in AI applications and data analysis to enable effective interdisciplinary collaboration and contribute to the design of AI solutions that address healthcare needs [37]. As nurses spend most time at bedside to provide care to patients, nurses play a crucial role in interpreting and responding to AI-generated risk assessments, such as those produced by fall prediction models. Comprehensive training empowers nurses to critically interpret AI outputs, building the trust necessary for seamless clinical integration.

### 4.2. Strengths and Limitations

Our fall prediction model considers more factors than the manual four-item assessment tool, and the results come from a broader perspective of views that may not be visible to humans. The development of this fall prediction model using local patient data represents a significant advancement in fall risk assessment within our hospital. This approach allows for a more nuanced and individualized prediction of fall risk, accommodating the exclusive epidemiological and clinical characteristics of our patient population. In addition, it was designed to integrate into our healthcare system, allowing automation of fall risk assessment in the daily clinic workflows of nurses. As such, it has a high potential to ease nurses’ workload without compromising patients’ safety. In our future work, we will involve other fall risk factors not already in our current database. For instance, adding fall history, a well-known risk factor, could improve the model’s accuracy in fall prediction. Other investigations, such as electrolyte disturbances and anemia, could also be included.

Several further limitations are noted. The 2016 dataset was extracted at the case level to avoid missing multiple fall events for the same patient, but the train/test split itself was subsequently confirmed by the author team to be patient-level: a patient with a prior fall is treated as a faller (using their earliest qualifying fall case), and any additional non-faller records for that same patient are excluded, so no patient contributes records to both the faller and non-faller groups, or to both the training and testing partitions. This mitigates the leakage risk raised during peer review [38] and is stated explicitly here for transparency. Non-faller controls were selected by simple random sampling rather than matched on demographic confounders (Section 2.3); age and sex were instead entered as model covariates. The 1:5 resampling ratio does not reflect the true population prevalence of inpatient falls (approximately 0.25–0.27%), and this was further compounded by a class_weight of {0:1,1:5} applied during model fitting; both can distort the calibration of predicted probabilities even where rank-based discrimination remains informative [39], so the reported AUC/sensitivity/specificity should not be read as calibrated absolute risk estimates for the true low-prevalence population. The first word-only matching rule used to flag high-risk medications (Section 2.3) could miss or mis-tag medications named differently across data sources or time periods. Exact hyperparameter search spaces are now reported (Table 4c), but per-variable missing-data proportions beyond those disclosed in Section 2.3 are not yet available. Finally, external validation beyond the single-center 2022–2023 cohort, and a fully labeled SVM coefficient table for the diagnosis and medication feature blocks (Section 4.1), remain priorities for the next revision. The following two further limitations, identified during an earlier field-testing phase of this model, remain relevant: new medications not yet indexed in the iPharm convention at the time of data extraction were not recognized by the feature-engineering pipeline, a challenge that could be mitigated by grouping medications at a therapeutic-class level rather than matching by individual generic name; medications are not currently grouped into any therapeutic-class hierarchy at all (individual cleaned names are matched directly), and introducing such a grouped hierarchy, whether at an internal iPharm level or a full ATC-equivalent classification, is planned for a future round (see Open Review 4, point 2); also, correlations between co-occurring diagnoses were not modeled, which could be addressed in future work using natural language processing or a similarity-matrix approach applied to diagnosis descriptions.

This study also has several limitations relating to external validity and generalizability. The model was developed and validated using data from a single tertiary hospital in Singapore, so performance in other institutions, healthcare systems, or patient populations is not yet established. Although the 2022 to 2023 validation cohort provides an important assessment of temporal drift, differences between the development and validation periods, including changes in clinical practice, medication formularies, coding systems, and patient case mix, may not fully represent the differences that would be encountered in other settings. External, multicenter validation involving different hospitals and electronic health record systems is required before the generalizability of this model can be established and is identified as a priority for future work.

Electronic health record integration across public hospitals in this country began in 2022 and remains ongoing. As this integration progresses, data from different institutions are expected to become more coherent and standardized, which may reduce some of the barriers to external validation and generalizability discussed above.

In addition to the discrimination measures reported in Section 3.5 and Section 3.6, model calibration was not formally assessed in this study. Calibration plots and the Brier score provide information on the agreement between predicted and observed risk that discrimination measures such as the area under the curve do not capture and are recommended additions for a future analysis of a future improved model [40]. Formal statistical comparison of the areas under the curve for the candidate models, for example, using the method described by DeLong and colleagues [41], was also not performed and is recommended for future work. This remains a limitation of the present study.

To support reproducibility, a detailed description of the data preprocessing, feature engineering, and recursive feature elimination pipeline is provided in Section 2.3 and Section 3.4, together with the complete grid search and hyperparameter configuration for each candidate model. The full list of retained diagnosis and medication features, and the complete set of model coefficients, is provided in the Supplementary Material. The implementation code is institutional property of the study hospital and is not publicly available. It may be made available upon reasonable request and subject to institutional approval from the hospital’s system administrators.

The implementation code for data preprocessing, feature engineering, and modeling pipeline is institutional property of the study hospital and is not publicly available. Access to the code may be granted upon reasonable request and is subject to institutional approval by the hospital’s system administrators. Nevertheless, detailed descriptions of the pipeline, grid search, hyperparameter configurations, and the complete list of features and model coefficients are provided in the main text and Supplementary Material to support reproducibility.

## 5. Conclusions

Our fall prediction model performed well in predicting falls in our retrospective dataset, suggesting its potential for validation and deployment as an automatic fall risk assessment tool. For the next phase, we intend to refine the model by exploring the classification of existing variables and adding new variables currently available in Datamart that may contribute to fall risk. With the development of this new capability and further AI machine learning, new possibilities can be explored, and the capability of the AI’s model expanded to enable the automation of other nursing assessments via AI.

## Figures and Tables

**Table 1:** Characteristics of prior research on developing artificial intelligence-assisted fall predictive model.

Authors	Sample Size/Population/Country	Dataset Split	Reduced Number of Features/Features Selected	Machine Learning Models	Best-Performing Model Based on AUC
Chu et al., 2022 [13]	1101/hospitalized older adults (>65 years old)/Taiwan	Iteration, and test sets in 3:1:1 ratio	21/instrumental activity of daily living, Braden score, activity of daily living, age, sex, weight, height, systolic pressure, diastolic pressure, visual analog scale, heart rate, communication disturbance, respiratory rate, mini nutritional assessment, polypharmacy, visual impairment, cardiovascular health study, sleep disturbance, hearing impairment, psychiatric medication, urinary incontinence	Deep neural network (DNN), extreme Gradient Boosting (XGBoost), Light Gradient Boosting Machine (LightGBM), random forest, Stochastic Gradient Descent (SGD) and logistic regression	XGBoost/ accuracy: 73.2% Sensitivity: 91.0% Specificity: 26.0% AUC: 57.0% F1 micro: 73.2% Precision micro: 72.1% Recall micro: 72.1%
Jung et al., 2019 [14]	15,480/adult inpatients/South Korea	Training and test sets in 4:1 ratio	12/sex, age, mental status, acuity score, length of stay, lower limb weakness, dysuria, fall prevention, services, use of restraints, type of admission route, diagnosis	Logistic regression, Cox proportional hazard regression, and decision tree	Logistic regression/ sensitivity: 79.03% Specificity: 79.43% PPV: 7.28 NPV: 99.46 AUC:85.9%
Lindberg et al., 2020 [15]	814/adult inpatients/United States	Training and 10-fold cross validation	38/sex, age, high-risk fall risk-increasing drugs, Morse Fall Scale, medical condition, hospital unit, Charlson Index, staffing information	Bagging, random forest, adaptive boosting, classification tree	Bagging/ sensitivity: 79.0% Specificity: 84.0% AUC: 89.0%
Liu et al., 2021 [16]	108,940/adult inpatients/Taiwan	Training and test sets in 2:1 ratio	54/demographics, admission diagnosis, self-care ability, physiological evaluation, surgical records, catheter records, focus charting, medication records, and fall scale score	Decision tree, Bayesian network, support vector machine, random forest (RF). Bagging and AdaBoost techniques were used to improve prediction performance	Bagging + RF Accuracy: 66.0% Sensitivity: 72.0% Specificity: 66.0% AUC: 76.0%
Oshiro et al., 2019 [17]	57,678/older adults (>60 years old)/United States	Training and validation set in 1.8:1 ratio	13/age, comorbidities, sex, other mental disorder, walking issues, Parkinson’s disease, urinary incontinence, depression, polypharmacy, psychotropic and anticonvulsant medications, osteoarthritis, osteoporosis	Logistic regression	Logistic regression/ sensitivity: 67.0% Specificity: 69.0% PPV: 8.0 NPV: 98.0 AUC: 74.0%

Notes: AUC = area under receiver operating characteristic curve.

**Table 2:** Data summary of fall and non-fall.

	Total Cases of Fall	Total Cases of Non-Fall	Fall + Control
**Number of unique case numbers**	251	101,410	101,661
**Number of unique cases matched with controls**	251	1255	1506

**Table 3:** Patients’ demographics (age and gender statistics).

Patient Characteristics	All Patients (*n* = 101,661)		Fallers (*n* = 251)		Non-Fallers (*n* = 101,410)	
	*n* (%)	Mean (SD)	*n* (%)	Mean (SD)	*n* (%)	Mean (SD)
Gender						
Male	51,806 (50.96)		155 (61.75)		51,651 (50.93)	
Female	49,855 (49.04)		96 (38.25)		49,759 (49.07)	
Age		54.86 (18.61)		61.67 (16.27)		54.84 (18.61)

**Table 4:** Feature reduction, dataset partitioning, and model hyperparameter selection. (**a**) Summary of feature reduction through recursive feature elimination (RFE). (**b**) Training and testing dataset. (**c**) Grid-search parameter space and final selected hyperparameters (Dataset D″, 325 features; 3-fold stratified cross-validation, F1-scored).

(**a**)					
**Variable**	**Before RFE** **(Dataset D)**	**After RFE** **(99.9%)** **(Dataset D′)**	**After RFE (95%)** **(Dataset D″)**	**Features** **(Incl. Surgery and Demo)**	**Importance** **(Sum)**
**Diagnosis**	1320	707	92	99%(D′); 95%(D″)	
**Medications**	1204	1025	216	99%(D′); 95%(D″)	
**Surgery**	15	15	15	Retained in all datasets	
**Demographics**	2	2	2	Retained in all datasets	
**Total Features**	**2539**	**1749**	**325**		
(**b**)					
	**Fallers**		**Non-Fallers**		**Total**
**Train**	175		878		1053
**Test**	76		377		453
(**c**)					
**Model**	**Grid Search Space**		**Final Selected Hyperparameters**		**CV F1**
Random Forest	n_estimators ∈ {50–490}; criterion = entropy (fixed); max_features ∈ {None, log2}; max_depth ∈ {4–8}; class_weight = {0:1,1:5} (fixed)		n_estimators = 60, max_depth = 5, max_features = log2		0.617
SVM	kernel ∈ {poly, rbf, sigmoid, linear}; C ∈ {0.01,0.1,1.0,2.0,5.0,10,50}; gamma = scale (fixed); class_weight ∈ {8 candidate weightings}		C = 0.01, kernel = linear, gamma = scale, class_weight = {0:1,1:5}		0.606
Decision Tree	class_weight ∈ {7 candidate weightings}; criterion ∈ {gini, entropy}; splitter ∈ {best, random}; max_features ∈ {sqrt, log2, None}; max_depth ∈ {4–8}		class_weight = {0:1,1:5}, criterion = gini, max_depth = 5, max_features = None, splitter = best		0.551
AdaBoost (DT base)	base_estimator = tuned decision tree (fixed); n_estimators ∈ {10–90}; learning_rate ∈ {0.05–1.0}		learning_rate = 0.5, n_estimators = 60		0.516

**Table 5:** Model results from the original dataset D (SVM row: sigmoid kernel outperformed the grid-search-recommended linear kernel on this full feature set; see Section 4.1).

Machine Learning Model	MCC	Negative Predictive Value	False Positive Rate	Precision = Positive Predictive Value	Specificity	Recall = Sensitivity	F1	AUC_ROC
SVM	0.489	0.955	0.210	0.440	0.790	0.816	0.571	0.803
RFDT	0.520	0.949	0.162	0.492	0.838	0.776	0.602	0.807
DT	0.428	0.937	0.212	0.412	0.788	0.737	0.528	0.762
LR	0.581	0.935	0.082	0.627	0.918	0.684	0.654	0.801
Ada Boost	0.363	0.913	0.191	0.395	0.809	0.618	0.482	0.714
GBDT	0.502	0.902	0.487	0.952	0.673	0.048		0.719

Note: AUC_ROC = area under the curve_receiver operating characteristic; MCC = Mathews Correlation Coefficient; SVM = support vector machine; RFDT = random forest decision tree; DT = decision tree; LR = logistic regression; AdaBoost = AdaBoost decision tree; GBDT = gradient boost decision tree.

**Table 6:** Results of 3 baseline models on dataset with RFE-selected feature D′.

Machine Learning Model	AUC	F1	Sensitivity/ Recall	Specificity	Precision	Positive Likelihood Ratio	Negative Likelihood Ratio
**SVM**	0.816	0.599	0.816	0.817	0.473	4.46	0.225
**Random Forests**	0.842	0.682	0.789	0.894	0.6	7.441	0.236
**Decision Trees**	0.778	0.574	0.711	0.846	0.482	4.62	0.342

Notes: AUC = 0.816; SVM = support vector machine.

**Table 7:** Results of 3 baseline models on dataset with RFE-selected feature D″ compared with manual fall risk assessments (SVM row: linear kernel—the final, deployed model; interpretability discussion in Section 4.1 refers to this model).

Machine Learning Model	AUC	F1	Sensitivity/ Recall	Specificity	Precision	Positive Likelihood Ratio	Negative Likelihood Ratio
SVM	0.800	0.573	0.803	0.798	0.445	3.98	0.247
Random Forests	0.798	0.567	0.803	0.793	0.439	3.88	0.483
Decision Trees	0.789	0.569	0.763	0.814	0.453	4.11	0.479
Hendrich II Fall Risk Model	0.667	-	0.750	0.513	0.950	1.540	0.490
NUH 4-item fall risk assessment	0.657	-	0.813	0.436	0.890	0.430	0.430

Notes: AUC = area under receiver operating characteristic curve; SVM = support vector machine; NUH = National University Hospital, Singapore.

## Data Availability

Data are available from the corresponding author on reasonable request, subject to applicable privacy and ethical retrictions.

## Supplementary Materials

The following supporting information can be downloaded at https://www.mdpi.com/article/10.3390/nursrep16080283/s1, File S1 TRIPOD Checklist: Prediction Model Development and Validation.

## Public Involvement Statement

No public involvement in any aspect of this research.

## Guidelines and Standards Statement

This manuscript was drafted against the Transparent Reporting of a multivariable prediction model (TRIPOD) and the Standards for Reporting Diagnostic Accuracy Studies (STARD) guidelines for predictive model development and diagnostic accuracy validation research.

## Use of Artificial Intelligence

Claude Sonnet 5 was used to assist with editing, restructuring, including reorganization of the methods, limitations and interpretability sections, correction of grammar and clarity, and reorganization of figures and tables for production. The AI tool was not used to generate, analyze, or fabricate any research data, results, or statistical findings. All data, analyses, and scientific conclusions presented in this manuscript are those of the human authors, who reviewed and verified all AI assisted edits before incorporation.

## Appendix A

Table A1 reports the complete set of model coefficients for the fall prediction model, ranked by absolute magnitude, referenced from Section 3.7.

**Table A1:** Top 30 SVM coefficients by magnitude, across all 325 features (full 325-row table available as Supplementary Material).

Feature	SVM Coefficient	Direction of Association
Ondansetron	+0.2829	+ (higher predicted risk)
Lactulose	+0.2814	+ (higher predicted risk)
Hirudoid	+0.2769	+ (higher predicted risk)
Quetiapine	+0.2768	+ (higher predicted risk)
Bisacodyl	+0.2748	+ (higher predicted risk)
S09—other/unspecified injuries of head	+0.2627	+ (higher predicted risk)
Hydroxyzine	+0.2502	+ (higher predicted risk)
Haloperidol	+0.2251	+ (higher predicted risk)
R55—syncope and collapse	+0.2200	+ (higher predicted risk)
Sennosides	+0.2147	+ (higher predicted risk)
R41—symptoms/signs involving cognitive functions	+0.2113	+ (higher predicted risk)
Potassium chloride	+0.2086	+ (higher predicted risk)
Alprazolam	+0.2052	+ (higher predicted risk)
Omeprazole	+0.1945	+ (higher predicted risk)
Mecobalamin	+0.1937	+ (higher predicted risk)
Ketoprofen	+0.1817	+ (higher predicted risk)
QV cream (emollient)	+0.1794	+ (higher predicted risk)
Morphine sulfate	+0.1792	+ (higher predicted risk)
Lynae (vitamin D/colecalciferol/calcium)	+0.1774	+ (higher predicted risk)
Promethazine codeine phosphate	+0.1731	+ (higher predicted risk)
Clopidogrel	+0.1704	+ (higher predicted risk)
Thiamine	+0.1700	+ (higher predicted risk)
Have_Risk_Drug (engineered)	+0.1698	+ (higher predicted risk)
Zolpidem tartrate	+0.1638	+ (higher predicted risk)
Vancomycin	+0.1635	+ (higher predicted risk)
Ticagrelor	-0.1600	− (lower predicted risk)
G62—other polyneuropathies	+0.1577	+ (higher predicted risk)
Sodium chloride	+0.1576	+ (higher predicted risk)
Lorazepam	+0.1511	+ (higher predicted risk)
Resource (nutritional supplement)	+0.1501	+ (higher predicted risk)

**Table A2:** SVM coefficients for the surgical-category and demographic feature blocks (confidently mapped from the documented training-pipeline column order).

Feature	SVM Coefficient	Direction of Association
Surgical category A	−0.028	− (lower risk)
Surgical category B	+0.062	+ (higher risk)
Surgical category C	−0.006	−
Surgical category D (Cardiovascular)	−0.085	−
Surgical category E	+0.040	+
Surgical category F (Digestive)	+0.011	+
Surgical category G	−0.010	−
Surgical category H (Male Genital)	0.000	none (0 fallers in category)
Surgical category I	−0.040	−
Surgical category J	−0.014	−
Surgical category K (Nervous)	−0.060	−
Surgical category L (Eye)	−0.010	−
Surgical category M	+0.006	+
Surgical category P	−0.078	−
Surgical severity (Ordinal, TOSP complexity)	−0.089	−
Age (scaled)	−0.013	−
Gender = male	+0.057	+ (male → higher predicted risk)

**Table A3:** Top 15 diagnosis categories by random forest importance (recursive feature elimination step; not SVM coefficients—category descriptions follow standard ICD-10 definitions and should be verified by the clinical author team).

Rank	ICD-10 Category	Standard Category Description	RF Importance
1	I61	Intracerebral hemorrhage	0.044
2	J38	Diseases of vocal cords and larynx, NEC	0.032
3	S01	Open wound of head	0.027
4	R11	Nausea and vomiting	0.025
5	S06	Intracranial injury	0.025
6	R41	Other symptoms/signs involving cognitive functions	0.025
7	S72	Fracture of femur	0.023
8	I95	Hypotension	0.022
9	I44	Atrioventricular and left bundle-branch block	0.021
10	R33	Retention of urine	0.021
11	R18	Ascites	0.020
12	J18	Pneumonia, organism unspecified	0.018
13	F62	Persistent personality changes NEC	0.018
14	I42	Cardiomyopathy	0.018
15	J69	Pneumonitis due to solids and liquids	0.017

**Table A4:** Top 15 medications by random forest importance (recursive feature elimination step; not SVM coefficients).

Rank	Medication/Engineered Feature	RF Importance
1	Have_Risk_Drug (engineered: ≥1 high-risk drug present)	0.082
2	No_of_Risky_Drug (engineered: count of high-risk drugs)	0.076
3	Lactulose	0.047
4	Omeprazole	0.044
5	Sennosides	0.039
6	Sodium chloride	0.034
7	Bisacodyl	0.032
8	Potassium chloride	0.028
9	Paracetamol	0.022
10	(unlabelled/missing medication name category)	0.017
11	Diben (nutritional supplement)	0.014
12	Hirudoid (topical anticoagulant cream)	0.014
13	Mupirocin	0.013
14	Lynae (vitamin D/colecalciferol/calcium)	0.012
15	QV cream (emollient)	0.012
