# Supplementary material for: Development and Validation of an Interpretable Machine Learning Model for Inpatient Fall Risk Using Electronic Health Record Data
Source: Nurs Rep. 2026 Aug 13;16(8):283. doi: 10.3390/nursrep16080283 (PMC13516745; doi:10.3390/nursrep16080283)
Supplement: Supplementary file 1 [file nursrep-16-00283-s001.zip › nursrep-4356521-supplementary.pdf]

TRIPOD Checklist: Prediction Model Development and Validation

Title: Development and Validation of an Interpretable Machine Learning Model for Inpatient Fall Risk Using Electronic Health Record Data				
**Section/Topic**	**Item**		**Checklist Item**	**Page**
**Title and abstract**				
Title	1	D;V	Identify the study as developing and/or validating a multivariable prediction model, the target population, and the outcome to be predicted.	Title page
Abstract	2	D;V	Provide a summary of objectives, study design, setting, participants, sample size, predictors, outcome, statistical analysis, results, and conclusions.	Abstract, Page 1
Introduction				
Background and objectives	3a	D;V	Explain the medical context (including whether diagnostic or prognostic) and rationale for developing or validating the multivariable prediction model, including references to existing models.	Page 2 to 4, Introduction, Table 1
	3b	D;V	Specify the objectives, including whether the study describes the development or validation of the model or both.	Page 4 to 5, and Abstract
Methods				
Source of data	4a	D;V	Describe the study design or source of data (e.g., randomized trial, cohort, or registry data), separately for the development and validation data sets, if applicable.	Section 2.1 and Section 2.3 Source of Data, Page 5
	4b	D;V	Specify the key study dates, including start of accrual; end of accrual; and, if applicable, end of follow-up.	Section 2.3 Source of Data, Page 17
Participants	5a	D;V	Specify key elements of the study setting (e.g., primary care, secondary care, general population) including number and location of centres.	Section 2.1, Page 5
	5b	D;V	Describe eligibility criteria for participants.	Section 2.4 Participants Page 5
	5c	D;V	Give details of treatments received, if relevant.	No
Outcome	6a	D;V	Clearly define the outcome that is predicted by the prediction model, including how and when assessed.	Section 2.5 Outcome Page 5
	6b	D;V	Report any actions to blind assessment of the outcome to be predicted.	Section 2.5 Outcome Page 6
Predictors	7a	D;V	Clearly define all predictors used in developing or validating the multivariable prediction model, including how and when they were measured.	Section 2.6 Predictors and Section 3.4 Page 6 and 9
	7b	D;V	Report any actions to blind assessment of predictors for the outcome and other predictors.	Section 2.6 Predictors Page 6
Sample size	8	D;V	Explain how the study size was arrived at.	Section 2.8 Sample Size
Missing data	9	D;V	Describe how missing data were handled (e.g., complete-case analysis, single imputation, multiple imputation) with details of any imputation method.	Section 2.7 Missing Data Page 7
Statistical analysis methods	10a	D	Describe how predictors were handled in the analyses.	Section 2.6 Predictors and Section 3.4 Page 6 and 9
	10b	D	Specify type of model, all model-building procedures (including any predictor selection), and method for internal validation.	Section 3.4 to 3.5, Page 9 to 13
	10c	V	For validation, describe how the predictions were calculated.	Section 3.6, Page 21 to 22
	10d	D;V	Specify all measures used to assess model performance and, if relevant, to compare multiple models.	Section 3.5 to 3.6 and Table 4 to Table 7
	10e	V	Describe any model updating (e.g., recalibration) arising from the validation, if done.	No
Risk groups	11	D;V	Provide details on how risk groups were created, if done.	No
Development vs. validation	12	V	For validation, identify any differences from the development data in setting, eligibility criteria, outcome, and predictors.	Section 4.1, Page 21 to 22
Results				
Participants	13a	D;V	Describe the flow of participants through the study, including the number of participants with and without the outcome and, if applicable, a summary of the follow-up time. A diagram may be helpful.	Figure 2, Page 6.
	13b	D;V	Describe the characteristics of the participants (basic demographics, clinical features, available predictors), including the number of participants with missing data for predictors and outcome.	Table 3, Page 8
	13c	V	For validation, show a comparison with the development data of the distribution of important variables (demographics, predictors and outcome).	Section 3.6, Page 12
Model development	14a	D	Specify the number of participants and outcome events in each analysis.	Table 2 and Table 4a, Page 6 and Page 13
	14b	D	If done, report the unadjusted association between each candidate predictor and outcome.	No
Model specification	15a	D	Present the full prediction model to allow predictions for individuals (i.e., all regression coefficients, and model intercept or baseline survival at a given time point).	Table 8, Page 18, Results Section 3.7
	15b	D	Explain how to the use the prediction model.	Section 3.7, Page 13
Model performance	16	D;V	Report performance measures (with CIs) for the prediction model.	Table 4 to Table 7 report point estimates Page 9, 11, 12
Model-updating	17	V	If done, report the results from any model updating (i.e., model specification, model performance).	No

**Discussion**				
Limitations	18	D;V	Discuss any limitations of the study (such as nonrepresentative sample, few events per predictor, missing data).	Section 4.2, Page 26 to 27
Interpretation	19a	V	For validation, discuss the results with reference to performance in the development data, and any other validation data.	Section 4.1, Page 21 to 22
	19b	D;V	Give an overall interpretation of the results, considering objectives, limitations, results from similar studies, and other relevant evidence.	Section 4.1, Page 15 to 22
Implications	20	D;V	Discuss the potential clinical use of the model and implications for future research.	Section 4.1 and Section 5, Page 22 and Page 28
**Other information**				
Supplementary information	21	D;V	Provide information about the availability of supplementary resources, such as study protocol, Web calculator, and data sets.	Section 4.2, Page 26
Funding	22	D;V	Give the source of funding and the role of the funders for the present study.	Funding section, Page 33

*Items relevant only to the development of a prediction model are denoted by D, items relating solely to a validation of a prediction model are denoted by V, and items relating to both are denoted D;V. We recommend using the TRIPOD Checklist in conjunction with the TRIPOD Explanation and Elaboration document.
